# Molluscum contagiosum multiples de l'enfant

**DOI:** 10.11604/pamj.2013.15.68.2566

**Published:** 2013-06-23

**Authors:** Hayat Bourra, Badreddine Hassam

**Affiliations:** 1 Service de Dermatologie, CHU Ibn Sina, Université Med V, Souissi, Rabat, Maroc

**Keywords:** Molluscums contagiosums, Sd de Job-Buckley, Déficit immunitaire primitif complexe

## Image en médecine

Le molluscum contagiosum est une tumeur cutanée bénigne d'origine virale. L'agent responsable est un Poxvirus à ADN fréquemment retrouvé chez les enfants de 3 à 16 ans, les immunodéprimés et plus rarement chez les adultes immunocompétents. Le syndrome d'hyper immunoglobulinémie E ou de Job-Buckley fait partie des déficits immunitaires primitifs complexes, caractérisé par l'association constante d'un taux élevé d'IgE, d'infections récidivantes pulmonaires et cutanées à staphylocoque, et de dermatite atopique. La coexistence de molluscums contagiosums lors de ce syndrome est rare, dans ce cas la multiplicité des lésions serait associée à l'immunodépression ou à la présence d'une dermatite atopique. Le meilleur traitement reste le curetage, mais au détriment de cicatrices disgracieuses. Chez les patients immunodéficients, la thérapeutique est souvent difficile et les récidives sont fréquentes, c'est pourquoi l'association de plusieurs techniques est préconisée. Nous rapportons le cas d'un enfant de 8 ans qui consulte pour des lésions papulo-nodulaires dont certaines ombiliquées, localisées au niveau du visage, du tronc, du dos et des membres supérieurs évoluant depuis 2ans faisant évoquer des molluscums contagiosums, confirmés à l'histologie. En reprenant l'interrogatoire avec la famille il existait un antécédent de tuberculose pulmonaire à 18mois, de multiples hospitalisations pour des bronchopneumopathies et dilatation de bronches, de dermatite atopique sans notion d'abcès cutanés et une hyperéosinophilie fluctuante faisant suspecter un Sd de Buckley. Le traitement des molluscums par cimétidine pendant 3 mois n'ayant pas donné de résultats, un traitement par isoprinosine associée à l'hydroxyde de potassium a été instauré avec persistance de quelques lésions traitées par curetage.

**Figure 1:**
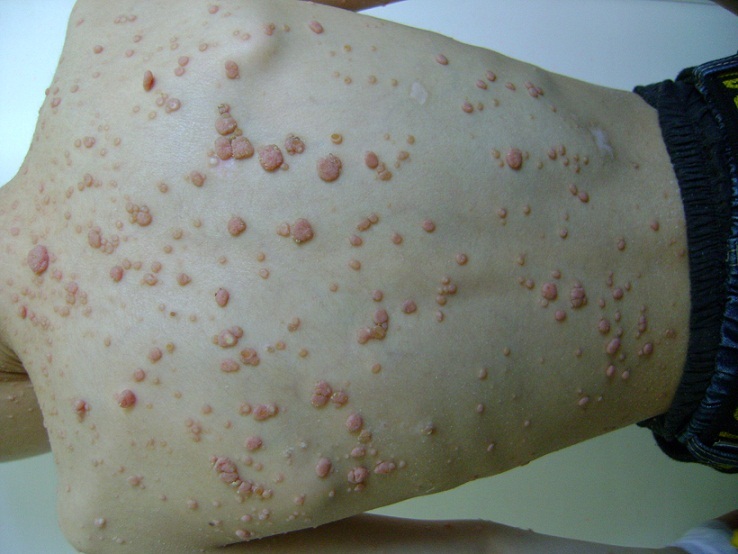
Papulo nodules blanc rosés, couleur chair, translucides, de tailles différentes, certains ombiliquées au centre siègeant au niveau du tronc

